# Successful Treatment of an Elderly Patient With Combined Small Cell Lung Cancer Receiving Anlotinib: A Case Report

**DOI:** 10.3389/fonc.2021.775201

**Published:** 2021-11-11

**Authors:** Yuying Gan, Pingli Liu, Tao Luo

**Affiliations:** Department of Respiratory Medicine, The Affiliated Hospital of Xuzhou Medical University, Xuzhou, China

**Keywords:** combined small cell lung cancer, elderly, anlotinib, long progression-free survival, the first-line therapy

## Abstract

Combined small-cell lung cancer (C-SCLC) is a relatively rare subtype of SCLC and is defined by the combination of SCLC and any elements of non-small-cell lung carcinoma. Anlotinib is a novel oral multitarget tyrosine kinase inhibitor that led to significant improvements in progression-free survival and overall survival in third-line therapy of advanced SCLC in the ALTER1202 study. Antiangiogenic therapy with anlotinib in C-SCLC has not previously been reported. An 80-year-old man was admitted with a 20-day history of blood-stained sputum. Chest computed tomography revealed a soft mass (45 × 43 mm) in the right upper lobe and a mediastinal lymph node and additional lung lesions in the homo lung. Pathology confirmed C-SCLC after an ultrasound-guided percutaneous puncture biopsy of the right lung tumor. The elderly patient was given anlotinib monotherapy at a dose of 10 mg/day on days 1–14 of a 21-day cycle after providing informed consent, and the outcome was assessed as continued partial response. As of the last follow-up evaluation, the patient’s progression-free survival was more than 7 months, and the treatment showed satisfactory safety. Our findings provide direct evidence of the efficacy of anlotinib in an elderly patient with C-SCLC. More studies are needed to confirm our observations.

## Introduction

Combined small-cell lung cancer (C-SCLC) is a subtype of SCLC according to the World Health Organization in 2015 (1). C-SCLC is defined as SCLC combined with any elements of non-small-cell lung cancer (NSCLC) and usually comprises squamous cell carcinoma, adenocarcinoma, large cell carcinoma, large cell neuroendocrine carcinoma, or any other rare components such as giant cell carcinoma or spindle-cell carcinoma. In previous studies, C-SCLC was found to account for 2%–28% of SCLC cases (2). The diagnosis rate with combined histology is higher in surgical specimens than those obtained by bronchoscopy or needle biopsy. The optimal treatments for C-SCLC have not been fully verified, which is often referred to as the SCLC (3).

SCLC proliferation has been demonstrated to be associated with the formation of microvessels, so inhibition of angiogenesis could be a promising treatment for SCLC (4). Anlotinib hydrochloride (AL 3818) is an oral tyrosine multiline inhibitor that targets the vascular endothelial growth factor receptor, platelet-derived growth factor receptor, fibroblast growth factor receptor, c-Kit, etc. The National Medical Products Administration approved anlotinib as a third-line treatment for SCLC in 2019 based on phase 2 of the ALTER1202 trial (5).

Here, we present a case report of an elderly patient with C-SCLC who achieved significant clinical benefit from anlotinib monotherapy as first-line therapy.

## Case Presentation

**Figure 1:**
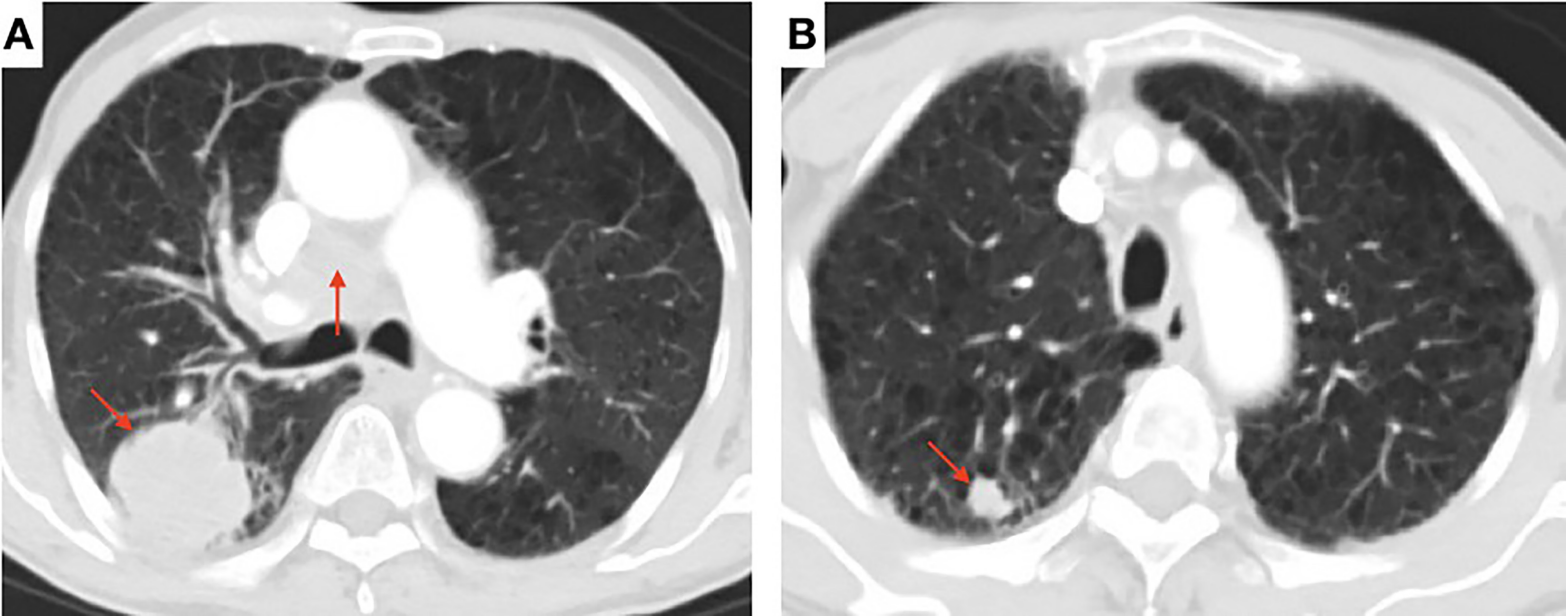
Contrast-enhanced chest computed tomography (CT) scan showing **(A)** a soft mass (45 × 43 mm) in the posterior segment of the right upper lobe and the enlarged right hilar and mediastinal lymph nodes (arrow) and **(B)** multiple pulmonary nodules with a maximum diameter of 20 × 14 mm (arrow).

**Figure 2:**
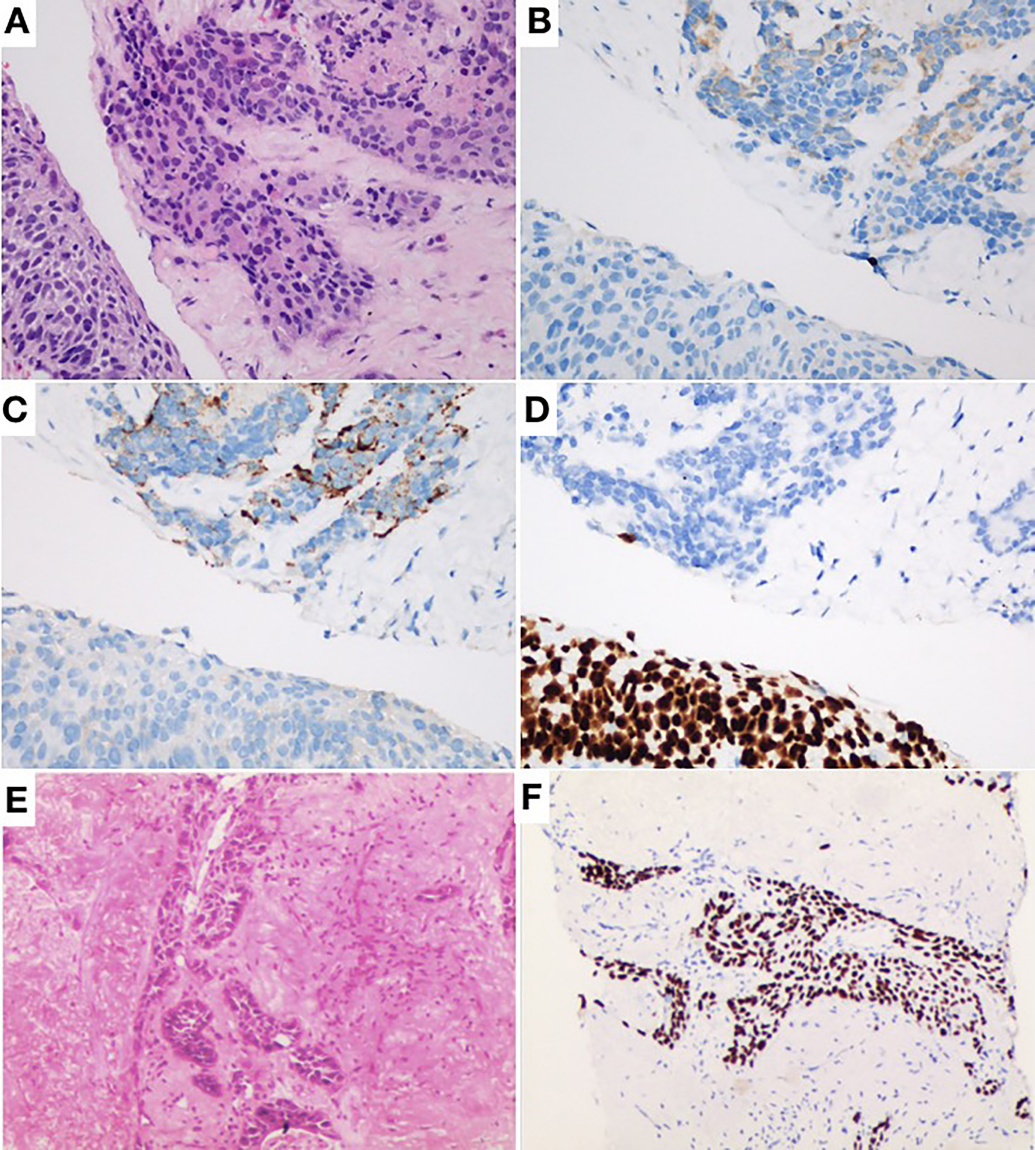
**(A)** Hematoxylin and eosin staining (H&E) × 40 showing combined small-cell lung cancer (small-cell carcinoma and squamous cell carcinoma). **(B)** Same field as in Panel **(A)** with positive cytoplasmic staining for synaptophysin as typical neuroendocrine. **(C)** Same field as in Panel **(A)** with positive cytoplasmic staining for chromogranin A as typical neuroendocrine. **(D)** P40 brown nuclear staining. **(E)** H&E × 10 showing infiltrative squamous cell carcinoma component. **(F)** Same field as in Panel **(E)** with unclear staining for P40.

**Figure 3:**
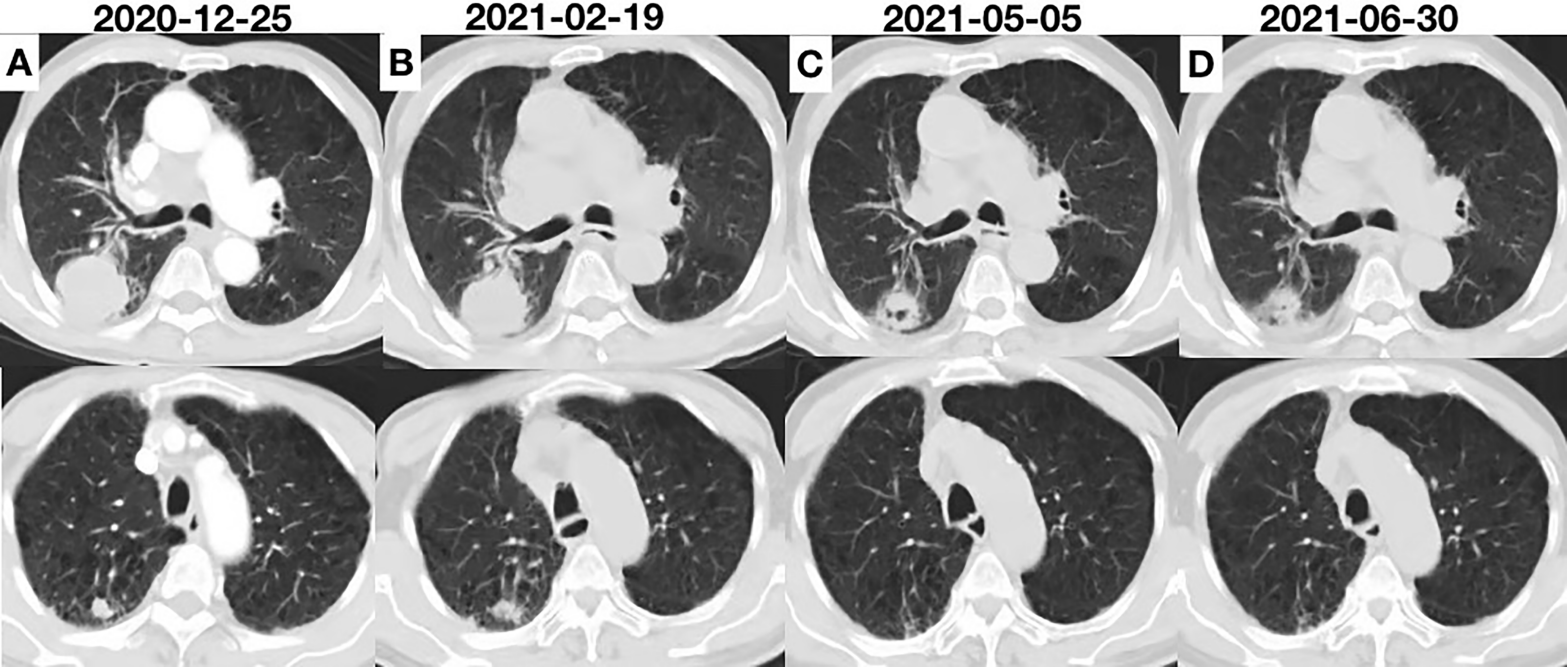
**(A)** Chest computed tomography (CT) scan showing the initial tumor. **(B)** Two cycles after anlotinib initiation; the efficacy was evaluated as SD. **(C)** CT scan showing remarkable shrinkage of the tumor; the efficacy was evaluated as PR. **(D)** CT scan showing sustained shrinkage of the tumor and a small amount of additional effusion; the efficacy was evaluated as PR.

A 80-year-old male former smoker with a Brinkman index of 400 was admitted to our department with a complaint of blood-stained sputum for 20 days on December 25, 2020. The patient had a history of chronic obstructive pulmonary disease. An enhanced computed tomography (CT) scan of the chest showed a mass (45 × 43 mm) in the posterior segment of the right upper lobe, multiple pulmonary nodules with a maximum diameter of 20 × 14 mm, and enlarged right hilar and mediastinal lymph nodes (**Figure 1**). An ultrasound-guided percutaneous puncture biopsy was performed against the right upper lobe mass, and the pathology was proved to be C-SCLC comprising SCLC and squamous cell carcinoma (**Figure 2**). The positron emission tomography scan for clinical staging showed significant uptake in each abnormal area, with the following standardized uptake values: posterior segment mass, 15 g/ml; posterior segment nodule, 9.0 g/ml; and right pulmonary hilar and mediastinal lymph nodes, 12.2 g/ml. The tumor–node–metastasis (TNM) stage was T2N2M1 IV according to the 8th edition of the American Joint Committee on Cancer (AJCC)/Union for International Cancer Control (UICC) TNM staging system for lung cancer. No epidermal growth factor receptor (EGFR) gene mutation was found in the biopsied tissue by next-generation sequencing. Considering that oral anlotinib therapy is generally better tolerated than conventional chemotherapy, after providing informed consent, the patient was treated with anlotinib (10 mg/day once daily orally, 2 weeks on and 1 week off). The patient was followed up with a chest CT in the outpatient department. The chest CT (February 19, 2021) showed a 39 × 39 mm mass in the right upper lobe and a 21 × 14 mm nodule in the right upper lobe, the lymph nodes in the right hilum and mediastinum were slightly enlarged, and the efficacy was evaluated as stable disease (SD) according to RECIST version 1.1. The patient continued to be treated with oral anlotinib monotherapy. Chest CT (May 5, 2021) showed a 30 × 30 mm mass in the right upper lobe, a 8 × 7 mm nodule in the right upper lobe, and significant shrinkage of the right pulmonary hilar and mediastinal lymph nodes; the curative effect was evaluated as partial response (PR). As a result of the reduction in mass, the patient was more confident in continuing with anlotinib. Chest CT from the last follow-up (June 30, 2021) showed a 23 × 22 mm mass in the right upper lobe, a 8 × 7 mm nodule in the right upper lobe, no significant changes in the right pulmonary hilar or mediastinal lymph nodes, and a small amount of additional unilateral pleural effusion; the efficacy was evaluated as PR (**Figure 3**). From the beginning of treatment to the last follow-up, the patient had been receiving anlotinib treatment for more than 7 months. During the treatment, the patient developed mild fatigue and hypertension. All these adverse events were defined as level 1.

## Discussion

C-SCLC is a rare subtype of SCLC with both components of SCLC and NSCLC. The incidence of C-SCLC has been previously reported to range from 2% to 28% in various studies. In our case, the patient was diagnosed as C-SCLC by hematoxylin–eosin staining and immunohistochemistry.

Owing to its rarity and complexity, only a few studies have focused on prognosis in C-SCLC. A previous study noted that there was no difference in overall survival (OS) between C-SCLC and pure SCLC in patients that did not receive surgery (6). In our case, the stage was too late for surgery to be suitable; the patient may have a poor prognosis without other effective treatments.

Treatment options are limited for elderly C-SCLC patients, who are often excluded from various clinical trials, leading to a lack of evidence-based medicine and insufficient clinical evidence for this group of patients. Moreover, there is no unified treatment for C-SCLC, referring to SCLC treatment, such as surgery, radiotherapy, and chemotherapy. In our case, the patient was 80 years old. Elderly patients are often thought to be too weak to tolerate treatment-related toxicity. However, in a recent study involving a total of 146 elderly SCLC patients aged from 80 to 92 years old, the median survival was 1.3 months without any treatment, 6 months with local therapy alone, 7.2 months with chemotherapy alone, and 14.4 months with chemotherapy plus local therapy (7). These results indicate that very elderly SCLC patients can benefit from aggressive multimodality therapy. Even so, the patient and his family in our case refused chemotherapy or radiotherapy because of his age. Targeted therapy and immunotherapy are generally better tolerated than conventional chemotherapy. According to previous studies, the likelihood of EGFR mutation in C-SCLC varies from 15% to 20% (8). Per some case studies, EGFR or ALK tyrosine kinase inhibitors may be effective (9). The patient was only tested for an EGFR mutation because of financial and healthcare concerns in this case; the results were negative for the EGFR gene. Atezolizumab and durvalumab improved OS in the first-line treatment of SCLC (10, 11), but not C-SCLC. A recent study showed that the failure of PD-L1 inhibitors in postoperative C-SCLC patients may be due to poor PS (12). The patient abandoned immunotherapy and refused to be tested for PD-L1 owing to the high cost and unproven efficacy of immunotherapy.

Anlotinib is a new tyrosine kinase inhibitor targeting a variety of factors, including tumor proliferation, the vascular system, and the tumor microenvironment. Anlotinib was shown to improve OS and progression-free survival (PFS) as a third-line therapy for SCLC patients, and the drug was approved in China. The efficacy and safety of anlotinib for elderly patients with previously treated extensive small-cell carcinoma was recently confirmed by a retrospective analysis (13). The study enrolled 79 elderly patients who were treated with anlotinib, with objective response rate of 8.9%, disease control rate of 69.6%, median PFS of 3.0 months, and median OS of 7.1 months. Importantly, most of the adverse reactions were grade 1–2. However, there are no reported cases of C-SCLC treated with anlotinib, and C-SCLC is excluded from trials in ALTER1202. In our case, the patient was given anlotinib at 10 mg once daily for 2 weeks on with 1 week off; the lesions shrank dramatically, and the treatment was well tolerated. Although a chest CT (June 30, 2021) revealed a tiny amount of further unilateral pleural effusion, the volume of pleural fluid was insufficient to determine the nature of the pleural effusion, and the patient continued to be treated with anlotinib. The patient was re-examined on September 27, 2021, with a chest CT indicating that the pleural effusion had been absorbed (**Supplementary Figure**), and the outcome was graded as maintained SD.

In our case, the patient was admitted to our department complaining of blood-stained sputum. Hemoptysis did not worsen after anlotinib therapy. The patient developed hypertension after anlotinib treatment. As shown in a recent study, hypertension that occurs during the delivery of anlotinib is associated with better prognosis (13). In addition, tumor cavitation was found to be an independent factor predicting better PFS (14). During anlotinib therapy, tumor cavitation occurred in our patient. However, effective predictive biomarkers of anlotinib are not clear. Further investigation is warranted.

## Conclusion

To the best of our knowledge, this is the first case of successful anlotinib treatment of an elderly patient with C-SCLC. Anlotinib is a potential treatment choice for elderly C-SCLC patients who refuse or cannot tolerate chemotherapy. More studies are needed to confirm our observations.

## Data Availability Statement

The original contributions presented in the study are included in the article/**Supplementary Material**. Further inquiries can be directed to the corresponding author.

## Ethics Statement

The studies involving human participants were reviewed and approved by the Ethics Committee of the Affiliated Hospital of xuzhou Medical University. The patient provided his written informed consent to participate in this study. Written informed consent was obtained from the individual(s) for the publication of any potentially identifiable images or data included in this article.

## Author Contributions

PL collected the patient’s clinical information. YG wrote and submitted the manuscript. TL revised and proofread the manuscript. All authors contributed to the article and approved the submitted version.

## Funding

The author received financial support from the Natural Science Fund for Colleges and Universities in Jiangsu Province (20KJB320011).

## Conflict of Interest

The authors declare that the research was conducted in the absence of any commercial or financial relationships that could be construed as a potential conflict of interest.

## Publisher’s Note

All claims expressed in this article are solely those of the authors and do not necessarily represent those of their affiliated organizations, or those of the publisher, the editors and the reviewers. Any product that may be evaluated in this article, or claim that may be made by its manufacturer, is not guaranteed or endorsed by the publisher.

## Supplementary Material

Supplementary Figure 1 Chest computed tomography (CT) scan showing that the pleural effusion had been absorbed, and the outcome was graded as maintained SD.
