# Supplementary figures and images for: Successful Treatment of an Elderly Patient With Combined Small Cell Lung Cancer Receiving Anlotinib: A Case Report
Source: Front Oncol. 2021 Nov 11;11:775201. doi: 10.3389/fonc.2021.775201 (PMC8632010; doi:10.3389/fonc.2021.775201)

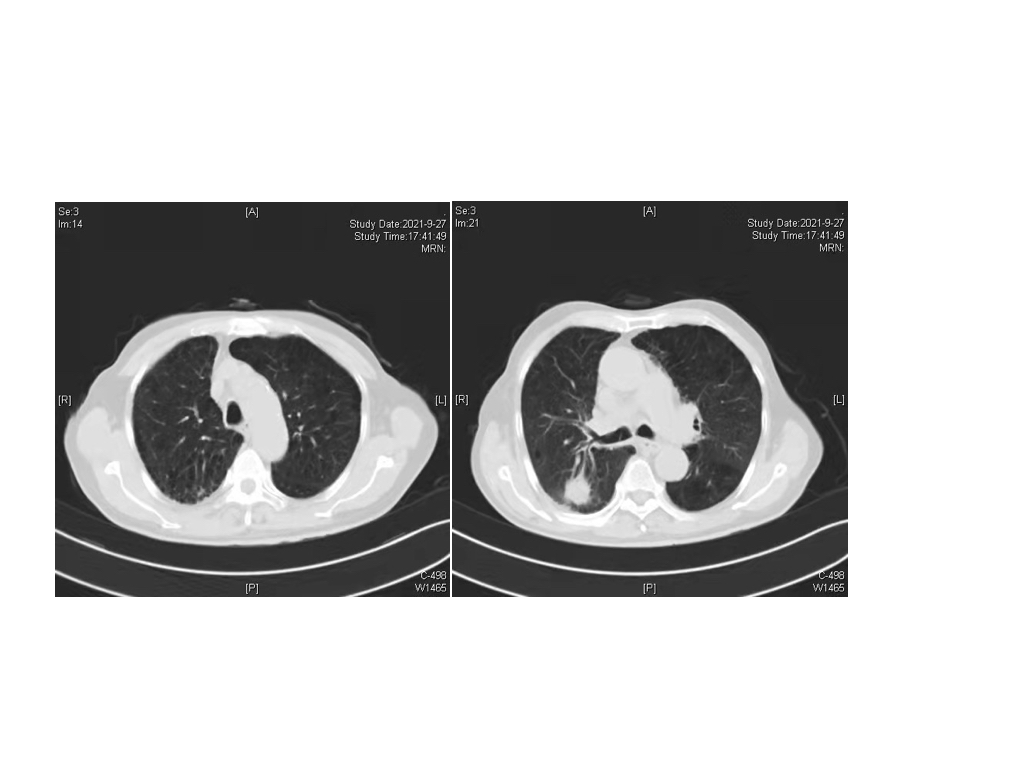

Supplement: Supplementary Figure 1 — Chest computed tomography (CT) scan showing that the pleural effusion had been absorbed, and the outcome was graded as maintained SD. [file Image_1.jpeg]
